# Diseases of the Eye

**Published:** 1904-09

**Authors:** 


Diseases of the Eye. Edited by L. Webster Fox, A. M., M. D., Professor of Ophthalmology in the Medico-Chirurgical College of Philadelphia, Pa.; Ophthalmic Surgeon in the Medico-Chirurgical Hospital. 584 pages, with five colored plates and two hundred and ninety-six illustrations in the text. New York: D. Appleton & Co. Price, $3.00.

The book is the outgrowth of a series of lectures delivered by the author at the Medico-Chirurgical College and Hospital, Philadelphia, during the last ten years. Among the articles of special importance is the treatment of albuminuric retinitis, hemorrhagic retinitis, lid operations, declinations of the retinal meridians, cataract, glaucoma, and diseases of the orbit. The illustrations are clear and very profuse, and the book is equally adapted to the use of the student or general practitioner. J.

